# The effect of space travel on human reproductive health: a systematic review

**DOI:** 10.1038/s41526-024-00351-1

**Published:** 2024-01-18

**Authors:** Marta Gimunová, Ana Carolina Paludo, Martina Bernaciková, Julie Bienertova-Vasku

**Affiliations:** 1 Department of Physical Activities and Health Sciences, Faculty of Sports Studies, Masaryk University, Brno, Czech Republic; 2 Department of Sport Performance and Exercise Testing, Faculty of Sports Studies, Masaryk University, Brno, Czech Republic

**Keywords:** Risk factors, Physiology

## Abstract

With increasing possibilities of multi-year missions in deep space, colonizing other planets, and space tourism, it is important to investigate the effects of space travel on human reproduction. This study aimed to systematically review and summarize the results of available literature on space travel, microgravity, and space radiation, or Earth-based spaceflight analogues impact on female and male reproductive functions in humans. This systematic review was performed according to Preferred Reporting Items for Systematic Reviews and Meta-Analyses guidelines and Space Biomedicine Systematic Review methods. The search was performed using three databases: PubMed, Web of Science, and Medline Complete. During the database search, 364 studies were identified. After the study selection process, 16 studies were included in the review. Five studies included female participants, and the findings show an increased risk of thromboembolism in combined oral contraceptive users, decreased decidualization, functional insufficiency of corpus luteum, and decreased progesterone and LH levels related to space travel or its simulation. Male participants were included in 13 studies. In males, reproductive health considerations focused on the decrease in testosterone and sex hormone-binding globulin levels, the ratio of male offspring, sperm motility, sperm vitality, and the increase in sperm DNA fragmentation related to space travel or its simulation. Results of this systematic review highlight the need to focus more on the astronaut’s reproductive health in future research, as only 16 studies were found during the literature search, and many more research questions related to reproductive health in astronauts still need to be answered.

## Introduction

To undertake multi-year missions in deep space, colonize other planets, and/or prepare appropriate safety measures for space tourism, it is important to investigate the possible effects of space travel on human reproduction. During space travel, astronauts are exposed to several hazardous factors, such as alterations in gravitation forces, including hypogravity and hypergravity, or ionizing radiation^1,2^. Exposure to microgravity has been demonstrated to impair the endocrine system in males^3^, muscle mass, and bone mass^4,5^; it also leads to altered fluid and electrolyte balance, cardiovascular changes, or increased glomerular filtration rate in both genders^2^. Experimental bed rest studies are typically used in humans to simulate spaceflight microgravity^6^. For in vitro samples, clinostat or random positioning machines are used to simulate microgravity by randomization of the gravity direction over time. In animal studies, hindlimb suspension is usually used to stimulate the physiological effects of microgravity^2^. Ionizing radiation, which is about 500 times greater in space compared to Earth conditions, was observed to cause DNA damage, apoptosis in ovarian follicles, and sperm DNA fragmentation in animal models^1,2,7,8^.

Until now, limited research has focused on the effect of space travel on the reproductive system and its function, along with endocrine regulation of reproduction or prenatal development. Endocrine regulation of sex hormones is the most investigated as it also impacts musculoskeletal health and skeletal muscle protein metabolism (e.g.^9,10^). Most of this research is based on animal models^1,2^.

Female mouse models show that microgravity affects embryonic stem cell growth and differentiation^11^, resulting in impaired decidualization of the endometrium needed for implantation and maintaining pregnancy^12^. Data from therapeutic radiation on ovaries suggests that space radiation exposure during a typical Mars mission may reduce the ovarian reserve by 50% by destroying some of the primordial follicles. Furthermore, space radiation may lead to a decreased time interval to menopause, leading to a decreased reproductive capacity of the female astronaut^13^. Exposure to total body radiation of 15 Gy leads to the loss of ovarian function in humans^14^.

In males, the microgravity exposure was observed to reduce the total sperm count in mice models^15^, decrease testes weight, and decrease testosterone concentrations in male rats^2,16^. Exposure to ionizing radiation increases sperm DNA fragmentation in *Echinogammarus marinus* models^7^, affecting the male reproductive health. Data from therapeutic radiation on testes in humans show that a dose higher than 1 Gy might result in azoospermia and risk for hereditary disorders^17^. Furthermore, decreased serum testosterone levels were observed in men treated with radiotherapy for rectal cancer when testes are exposed to direct or scattered radiation^18^.

As described in *The Impact of Sex and Gender on Adaptation to Space: A NASA Decadal Review*^19^, reproductive demographics of female and male US astronauts significantly differ based on biological processes and gender roles for parents. Women are usually the primary caregivers (e.g., ref. ^20^) and are often required to take an extended family leave from their career when having a child (e.g., ref. ^21^). A smaller number of female astronauts (44.7%) have at least one child compared to the male astronauts (83.9%). Female astronauts also significantly delay reproduction, on average, by 5.6 years compared to males. It was hypothesized that the delayed reproduction in female astronauts is related to the required extensive space travel training time^19^. A new NASA decadal review is expected next year, adding more current data on the topic. However, not only delayed reproduction but also the impact of the potential acceleration of aging and gonadal radiation exposure related to space travel might be other factors affecting the reproduction capacity in female astronauts^19^. Therefore, the aim of this study was to systematically review and summarize the results of available literature on space travel, microgravity, and space radiation’s impact on female and male reproductive functions in humans.

## Results

### Characteristics of included studies

During the database search, 364 studies were identified (Pubmed: 121 articles; Medline Complete: 142 articles; Web of Science: 101 articles). After the duplicate removal (*n* = 160), studies involving animal samples (*n* = 45), different methodologies, or non-English language articles (e.g., conference paper, book chapter; *n* = 12), 147 studies were screened based on title and abstract in which 120 studies were excluded. In the last stage, 27 studies remained, followed by the exclusion of 5 studies due to no full-text being available and eight studies because the outcomes needed to match the topic. Via another method (accidental find), two studies focusing on ionizing radiation’s effect on male reproductive health were found^22,23^. A total of 16 studies were included in the systematic review.

The methodological quality of the included studies (Table 1) ranged from 70.0%^24^ to 90.9%^25^, suggesting good methodological quality^26^. The most common methodological deficits consisted of not reporting the study’s hypothesis clearly, not reporting the probability values, and the lack of representativeness of the source population. The methodological quality of the included studies is shown in Supplementary Table 1.

**Table 1:** Final score of methodological quality of included studies.

Classification	Final score	Study
Good methodological quality	70.0%	Gorbacheva et al.^24^
	72.7%	Cho et al.^12^; Zhou et al.^23^; Strollo et al.^27^
	80.0%	Ikeuchi et al.^31^; Liang et al. ^28^; Belavy et al.^6^; Smorawinski et al.^29^
	81.,8%	Kumar et al.^22^; Tomilovskaya et al. ^32^; Little et al.^33^; Zwart et al.^34^
	88.9%	Boada et al.^35^
	90.0%	Zachwieja et al.^10^; Smith et al.^30^
	90.9%	Loder et al.^25^

Six of the included studies used bed rest study design. Bed rest studies’ methodological quality (Table 2) ranged between 2^27^ to 7 points^28^. No study indicated a prohibition of sunlight exposure. Four studies did not indicate a set wake/sleep time^6,10,29,30^. One study design allowed a limited ambulation time (to shower and toilet)^29^. In three of the included studies, the recommended 6° head-down tilt was not used^6,27,29^.

**Table 2:** Bed rest studies transferability.

	Strollo et al.^27^	Smith et al.^30^	Belavy et al.^6^	Liang et al.^28^	Smorawinski et al.^29^	Zachwieja et al.^10^
6° head-down tilt	x	✓	x	✓	x	✓
Individualised and controlled diet	?	?	✓	✓	✓	✓
Set a daily routine with fixed wake/sleep time	x	?	?	✓	?	?
Bed rest phases are standardized for all participants.	?	✓	✓	✓	✓	✓
Uninterrupted bed rest except for test condition	✓	?	✓	✓	x	✓
Sunlight exposure prohibited	?	?	?	?	?	?
All measures were taken on the same day and time.	?	?	✓	✓	✓	?
Bed rest duration	1 hour	60 or 90 days	56 days	45 days	3 days	28 days
Total points	2	3	5	7	4	4

Articles meeting the inclusion criteria included four articles from USA (25%), two articles from China (12, 5%), two articles from Russia (12, 5%), one article from Poland (6, 25%), one article from Italy (6, 25%), one article from South Korea (6, 25%), one article from India (6, 25%), one article from Japan (6, 25%), one article from Spain (6, 25%), one article from Austria (6, 25%), and one article from Germany (6, 25%)^6,10,12,22–25,27,28,29–35^.

As shown in Table 3, in most of the included studies, only male participants were analyzed (11 studies; 68, 75%^6,10,22,23,25,28,29–31,33,35^), three studies focused on female participants (18, 75%^12,24,32^), and two studies included a mixed sample (12, 5%^27,34^).

**Table 3:** Overview of measured outcomes of the 16 included studies.

Participants	Study setting	Main outcome	Study
Female	Earth (dry immersion)	menstrual cycle, endocrine parameters	Gorbacheva et al.^24^
Female	Earth (dry immersion)	menstrual cycle	Tomilovskaya et al.^32^
Female	Earth (clinostat)	endometrial stromal cells	Cho et al.^12^
Mixed sample	Earth (head-down bed rest)	endocrine parameters	Strollo et al.^27^
Mixed sample	Space	venous thromboembolism risk	Zwart et al.^34^
Male	Earth (parabolic flight)	sperm	Boada et al.^35^; Ikeuchi et al.^31^
Male	Earth (ionizing radiation occupational exposure)	sperm	Kumar et al.^22^; Zhou et al.^23^
Male	Earth (head-down bed rest)	endocrine parameters	Liang et al.^28^; Smith et al.^30^; Zachwieja et al.^10^
Male	Earth (bed rest)	endocrine parameters	Belavy et al.^6^; Smorawinski et al.^29^
Male	Earth (water submersion)	endocrine parameters	Loder et al.^25^
Male	Space	endocrine parameters	Smith et al.^30^
Male	Space	offspring sex ratio	Little et al.^33^

Thirteen articles reported data from the experiments on Earth. Simulated microgravity by parabolic flight was used in two studies^31,35^ and by clinostat system in one study^12^; in two studies ionizing radiation occupational exposure was used^22,23^, head-down bed rest was performed in four studies^10,27,28,30^, bed rest was conducted in two studies^6,29^, in two studies dry immersion was used^24,32^, and in one study data from water submersion were reported^25^. Three articles reported data on space exposure^30,33,34^. Eight of included studies focused on endocrine changes after the space flight or its simulation^10,25,6,27,28,29,30^; four studies focused on sperms^22,23,31,35^; two studies focused on menstrual cycle changes^24,32^; one study focused on endometrial stromal cells^12^, one study focus of the venous thromboembolism risk^34^, and one study focused on offspring sex ratio in male astronauts^33^.

### Female and mixed studies

Three studies, including female participants, and two studies, including mixed samples, were identified during the study selection process. Their characteristics are shown in Table 4. One study used a clinostat system to simulate the microgravity of in vitro samples of human endometrial stromal cells (PSCs) from the uterus^12^. Two studies used dry immersion to simulate microgravity; in both studies, participants were allowed 30 min/day to spend outside the immersion bath for hygiene procedures^24,32^. Tomilovskaya et al.^32^ described the first female dry immersion study, and their participants were involved in their study for two menstrual cycles. The three-day dry immersion occurred between day 7 and day 10 of participants’ menstrual cycles^32^. In a study by Gorbacheva et al.^24^, two menstrual cycles were followed; the 5-day-long dry immersion was performed between day 10 and day 15 of participants’ menstrual cycles. One study used one hour-long -12° head-down bed rest to simulate microgravity and analyze endocrine parameter changes^27^. In one study, spaceflight exposure was used to analyze proteins involved in clotting cascade from blood samples obtained twice before spaceflight, five times during the flight, and twice after flight^34^.

**Table 4:** Female and mixed studies participants’ characteristics and study settings.

Author (year)	Country	Study	Sample size	Gender	Age range (years)	Type of Exposure	Time of Exposure	in vivo/
		setting						in vitro
Zwart et al.^34^	USA	Space	13	females*	F: 43 ± 4	spaceflight	up to 180 days	in vivo
					(mean ± SD)			
			52	males	M: 48 ± 5			
					(mean ± SD)			
Strollo et al.^27^	Italy	Earth	6	females	24 to 35	−12° head-down bed rest	1 h	in vivo
			6	males				
Cho et al.^12^	Republic of Korea	Earth	25	females	40 to 45	clinostat system (5 rpm)	36 h	in vitro
Tomilovskaya et al.^32^	Russia	Earth	6	females	24 to 39	dry immersion	3 days	in vivo
Gorbacheva et al.^24^	Russia	Earth	12	females	22.7 to 40.8	dry immersion	5 days	in vivo

^*^8 users of combined oral contraceptives, 5 non-users of combined oral contraceptives; rpm, rotations per minute.

In Table 5, results from female and mixed samples studies are shown. In female in vitr*o* samples, exposure to microgravity was observed to decrease decidualization (the process of endometrial cells in preparation for, or during pregnancy) by decreasing proliferation and migration and endometrial stromal cells growth rate through Akt/MMP and FOXO3a/autophagic flux^12^. Two studies reported that menstrual cycle length stayed intact after dry immersion exposure^24,32^. Gorbacheva et al.^24^ observed decreased luteinizing hormone (LH), progesterone, and ovarian volume at day 9 of the menstrual cycle after the immersion. On the other hand, an increase in dominant follicle diameter and no change in uterus size and endometrial thickness were reported^24^. The mixed sample study focused on venous thromboembolism risk in male and female astronauts, showing an increased risk in females taking combined oral contraceptives^34^. A head-down bed rest mixed sample study reported no statistically significant change in oestradiol, testosterone, and LH levels after the rest^27^.

**Table 5:** Female and mixed studies’ results.

Author (year)	Study setting	Venous thromboembolism risk	Decidualization	eSCs growth rate	Oestradiol	Testosterone	Progesterone	LH	FSH	MC length	Uterus	Ovary
Zwart et al.^34^	Space	↑ cOCs users										
Strollo et al.^27^	Earth				≈	≈		≈				
Cho et al.^12^	Earth			↓	↓							
Tomilovskaya et al.^32^	Earth									≈		
Gorbacheva et al.^24^	Earth						↓	↓	≈	≈	≈	↓ volume, ↑ dominant follicle diameter

↓, statistically significant decrease; ≈, no statistically significant difference; ↑, statistically significant increase; *MC* menstrual cycle, *cOCs* users, combined oral contraceptives users; *LH* luteinizing hormone, *eSCs* endometrial stromal cells.

### Male studies

Eleven studies focused on male participants were identified during the study selection process. In Table 6, the male studies’ characteristics are summarized. In the study by Little et al.^33^, retrospective data from astronauts’ biographies were included in the analysis of offspring ratio. A study by Smith et al.^30^ focused on the effect of long- and short-duration space flight and −6° head-down bed rest on testosterone levels. In two studies, parabolic flights were used to simulate short-duration microgravity^31,35^. In a study by Boada et al.^35^, twenty parabolic flight maneuvers (8.5 s of microgravity for each parabola) were used for frozen sperm samples. In a study by Ikeuchi et al.^31^, fresh sperm samples underwent ten parabolic flight maneuvers (20 to 25 s of microgravity for each parabola). Occupational low-dose exposure to ionizing radiation while working with radiation in a hospital on sperm characteristics was analyzed in two studies^22,23^. Four studies analyzed the effect of bed rest or −6° head-down bed rest on testosterone levels^10,6,28,29^. Studies by Belavy et al.^6^, Liang et al.^28^, and Zachwieja et al.^10^ applied a strict bed rest. Study design by Smorawinski et al.^29^ provided 20 min/day to ambulate (to shower and toilet). In one study, the effect of water submersion on testosterone levels was analyzed^25^. In the study by Loder et al.^25^, divers were allowed to emerge for less than 20 min every 4 hours to urinate, defecate, drink, or undergo medical checks.

**Table 6:** Male studies participants’ characteristics and study settings.

Author (year)	Country	Study	Sample size	Gender	Age range (years)	Type of Exposure	Time of Exposure	in vivo/
		setting						in vitro
Little et al.^33^	USA	Space	18	males		spaceflight		in vivo
Smith et al.^30^	USA	Space	15	males	46 ± 4	long duration international space station expedition	48–215 days	in vivo
					(mean ± SD)			
		Space	9	males		short duration Space Shuttle mission	12–13 days	in vivo
		Earth	8	males		−6° head-down bed rest	60–90 days	in vivo
Boada et al.^35^	Spain	Earth	15	males	26 to 40	parabolic flight of frozen sperm samples	20 parabolic manoeuvres	in vitro
Ikeuchi et al.^31^	Japan	Earth	18	males	27.4 ± 5.4	parabolic flight of fresh sperm samples	10 parabolic manoeuvres	in vitro
					(mean ± SD)			
Kumar et al.^22^	India	Earth	83	males	27.7 ± 0.8	ionizing radiation occupational exposure	1+ years of low-dose radiation	in vivo
					(mean ± SD)			
Zhou et al.^23^	China	Earth	46	males	28.3 ± 3.1	ionizing radiation occupational exposure	2+ years of low-dose radiation	in vivo
					(mean ± SD)			
Belavy et al.^6^	Germany	Earth	10	males	33.4 ± 6.6	bed rest	56 days	in vivo
					(mean ± SD)			
Liang et al.^28^	China	Earth	8	males	26.1 ± 4.1	−6° head-down bed rest	45 days	in vivo
					(mean ± SD)			
Smorawinski et al.^29^	Poland	Earth	32	males	21.7 ± 1.5	bed rest	3 days	in vivo
					(mean ± SD)			
Zachwieja et al.^10^	USA	Earth	4	males	31 to 47	−6°head-down bed rest	28 days	in vivo
Loder et al.^25^	Austria	Earth	4	males	46.5 ± 0.9	water submersion, diving depth 2.5 m	41 h	in vivo
					(mean ± SD)			

One of the studies including male participants in the space study setting (Table 7) focused on the offspring sex ratio, showing a decreased ratio of male offspring (43.75%) in male astronauts^33^. Furthermore, the study by Little et al.^33^ observed a decreased male offspring ratio of 38.41% in high G pilots compared to 50.34% in low G pilots. The second study using the space study setting focused on endocrine changes, showing no statistically significant changes in testosterone and sex hormone-binding globulin (SHBG) during or after the short and long-duration space flight. A decrease in total, free, and bioavailable testosterone was observed only on the landing day after the space flight, probably as the transient effect of flight^30^. One study observed decreased sperm motility after microgravity exposure^31^, and another study by Boada et al.^35^ observed no statistically significant change in sperm motility, vitality, or sperm DNA fragmentation after exposure to microgravity. Occupational ionizing radiation exposure was observed to decrease sperm motility, vitality, and concentration and to increase sperm DNA fragmentation in comparison with non-exposed controls^22,23^. Bed rest and head-down bed rest studies show no statistically significant change in testosterone and prolactin after the rest^10,28,29,30^. SHBG was observed to decrease after the bed rest^6^. After the water submersion, a decrease in plasma testosterone was observed^25^.

**Table 7:** Male studies’ results.

Author (year)	Study setting	SHBG	Testosterone	Prolactin	Offspring sex ratio	Sperm motility	Sperm vitality	Sperm concentration	Sperm DNA fragmentation
Little et al.^33^	Space				↓ males (43.75%)				
Smith et al.^30^	Space	≈	≈ during the flight, ↓ on landing day						
	Space		↓ after the flight						
	Earth		≈ during the BR, ↓ 7 days before and 5 days after the BR						
Boada et al.^35^	Earth					≈	≈		≈
Ikeuchi et al.^31^	Earth					↓			
Kumar et al.^22^	Earth					↓	↓		↑
Zhou et al.^23^	Earth					↓		↓	↑
Belavý et al.^6^	Earth	↓ after BR	↑ in the first 3 weeks, ≈ after BR	≈ after BR					
Liang et al.^28^	Earth		≈						
Smorawinski et al.^29^	Earth		≈						
Zachwieja et al.^10^	Earth		≈						
Loder et al.^25^	Earth		↓						

↓, statistically significant decrease; ≈, no statistically significant difference; ↑, statistically significant increase; *BR* bed rest, *SHBG* sex hormone-binding globulin.

## Discussion

The aim of this study was to systematically review and summarize the results of available literature on space travel, microgravity, and space radiation impact on female and male reproductive functions in humans. The reproductive health consideration of space travel differs for female and male astronauts. In female astronauts, they include oral contraceptive use^34^, progesterone and LH levels^27^, ovarian and uterus changes^24^, decidualization, and endometrial stromal cell growth rate^12^. In males, reproductive health considerations focus on testosterone and SHBG levels^10,25,6,27,28,29,30^, the ratio of male offspring^33^, sperm motility^22,23,31,35^, sperm vitality^22,35^, and sperm DNA fragmentation^22,23,35^. To support those considering these options, it might be helpful to explore assisted reproductive technologies such as oocyte and sperm cryopreservation, along with reproductive counseling possibilities, as suggested by Rose^13^ and Ronca et al.^36^.

In female astronauts, the endocrine regulation of the menstrual cycle involves the hypothalamic release of gonadotropin-releasing hormone, which stimulates the pituitary gland to produce follicle-stimulating hormone and luteinizing hormone, which peaks mid-cycle and invokes ovulation^37^. The developing ovum in ovaries produces estrogen, and *the corpus luteum*, which forms after ovulation, produces progesterone. Animal models show a decrease in luteinizing hormone related to 37 days-long spaceflights; however, no changes in estrous cycle stages were observed^38^. In naturally cycling women, simulated microgravity by dry immersion led to a decrease in luteinizing hormone by 12% and progesterone by 52%, showing functional insufficiency of *corpus luteum*^24^. The menstrual cycle length was not altered after 3 and 5 days of dry immersion^24,32^. One hour of −12° head-down bed rest did not induce any significant changes in the endocrine regulation of the cycle, suggesting that longer microgravity exposure is needed to affect the endocrine regulation of the menstrual cycle^27^. Despite the fact that abnormal uterine bleeding is a common complaint among reproductive-aged women^39^, uterine bleeding changes were not analyzed in any of the included studies.

As the menstrual bleeding flow management during space flight training and the space flight can be challenging, medically induced amenorrhea using combined oral contraceptives is often used by female astronauts^40^. However, combined (progestin and estrogen) oral contraceptives were associated with lower circulating concentrations of albumin, higher concentrations of transferrin, and elevated markers of inflammation, which can contribute to an increased risk of venous thromboembolism event during space travel^34^. The occlusive deep venous thrombosis was diagnosed in one female astronaut during a long-duration spaceflight^41^, highlighting the need to carefully consider the type of combined oral contraceptives used before and during flight^34^.

Human pregnancy is currently contradicted during space flight as a safety measure to protect the fetus^13,42,43^. Multi-year duration space flights and colonization will require understanding the impact of space flight on pregnancy, and simulation studies will try to provide better insight into reproduction in space. Fetal development, long-term effects on gestation under space conditions, and monitoring the development and function of offspring conceived and developed in space are some of the potential priorities for future space programs as described in a European perspective of human development and reproduction in space by Jain et al.^43^. The study by Cho et al.^12^ showed that exposure to simulated microgravity leads to decreased decidualization and endometrial stromal cells growth rate due to decrease in Akt activity and FOX03a expression leading to an unreceptive endometrium. Furthermore, if microgravity and space radiation alter the pro-oxidant/antioxidant balance during pregnancy, it can increase the risk of miscarriage, preterm birth, or fetal growth restriction^44^. The absence of gravitational loading during the last trimester of gestation may cause hypotrophy of muscles and osteopenia in the trunk and legs, leading to delayed acquisition of developmental milestones such as sitting or walking of the fetus developed in space^45^. Animal models show increased perinatal morbidity for the rats that spent 9 to 20 days in spaceflight during their gestation. In surviving offspring, no delay in walking acquisition was observed^46^.

High-altitude airplane flights, e.g., transatlantic flights, constitute trivial cosmic radiation exposure for casual travelers. Pregnant pilots, flight attendants, and frequent flyers may exceed the recommended radiation exposure^47^. During transatlantic air travel in the third trimester of pregnancy, most of the pregnant women report no change in fetal movements during take-off or flight^48^. A study by Grajewski et al.^49^ focusing on miscarriage risk among flight attendants shows that cosmic radiation exposure of 0,1 mGy or more may be associated with an increased risk of miscarriage in weeks 9 to 13. However, the miscarriage risk was also associated with other factors such as work during sleep hours and high physical demands, and the miscarriage risk was not increased among flight attendants compared to a control group of teachers^49^. Maternal stress and exposure to stressful events during pregnancy were observed to impact the infant’s physical health^50^, premature birth, and low birth weight^51^, suggesting a possible negative effect of space travel-related stress on the fetus.

Space travel may increase the carcinogenic risk to reproductive organs. This risk was proposed to be higher in women as they have a higher incidence of radiation-induced cancers, as widely discussed in Market al.^19^. Still, the low number of female astronauts does not allow for assessment of the risk of spaceflight on gynecological cancer^36^.

In expert opinion by Rose^13^, significantly reduced ovarian reserve and consequent decrease in the reproductive capacity and decreased time interval to menopause caused by space radiation was suggested in female astronauts. Unfortunately, no original article showing the data about reproductive capacity or age of menopause in astronauts was found during the literature search in this systematic review.

Testosterone is the key hormone in the development of the male reproductive system and promotes muscle and bone mass^52^. Testosterone has been, therefore, often considered as a potential countermeasure for musculoskeletal losses related to space flight (e.g.^10^). The testosterone level seems unchanged by the space flight or bed rest study settings^9,10,6,27,28,30^ apart from the transient effects after flight^30^. A decrease in testosterone levels was observed in a short-term water submersion (41 h) study by Loder et al.^25^. Similarly, it was hypothesized that the decrease is related to stress effect^25^. The self-rated sexual drive was reported to temporarily decrease during space flight in male astronauts parallelly to urinary, plasma, and salivary testosterone levels in a study by Strollo et al.^53^. Similarly, animal studies show a decrease in testosterone levels in simulated microgravity studies caused by a reduction in testicular blood flow related to body fluid shift^1^.

Prolactin and LH levels did not change during the analog bed rest study^27,6^. Similarly, no LH and FSH levels change was observed after a 6-week hindlimb suspension in animal models^54^. Serum SHBG levels were observed to decrease during bed rest in inactive participants. The physical activity load during the bed rest led to stable SHBG levels^6^. Similarly, no change during or after the space flight in the level of SHBG was observed by Smith et al.^30^

Results observed by Ikeuchi et al.^31^ using fresh semen suggest that sperm motility is reduced by microgravity. In a study by Boada et al.^35^ using frozen semen, no significant change in sperm motility, vitality, or sperm DNA fragmentation was observed compared to Earth condition after a similar parabolic flight experiment as used by Ikeuchi et al.^31^. These results suggest that the sperm integrity may be protected by cryopreservation during the space flight when transporting male human gametes into space^35^. Still, chronic occupational exposure to ionizing radiation was observed to have a detrimental effect on sperm motility, vitality, concentration, and DNA fragmentation^22,23^. Similarly, ionizing radiation and microgravity were observed to increase sperm DNA fragmentation in animal studies^1^. Furthermore, a decreased sex ratio of male offspring by male astronauts exposed to high G forces was reported by Little et al.^33^. The authors hypothesized that sperm sex differences in sperm motility and longevity, smaller size, and cytoplasm content in Y sperm were the reason of decreased sex ratio of male offspring as higher G forces may accelerate metabolism in sperm subtracting energy available for travel to the ovum^33^. However, current knowledge shows no morphological differences between X and Y sperms in humans^55^. Still, X and Y sperms differences in genetic content may lead to differences in their stress response^56^. The study by You et al.^57^ reported that the viability of human Y spermatozoa was lower after exposure to stress (e.g., different temperatures and culture periods) compared to X spermatozoa, which may result in a shift of the offspring sex ratio as observed by Little et al.^33^. Similarly, low male sex offspring ratio associated with occupational testicular radiation exposure was observed in a previous study^58^. On the other hand, no association between offspring sex ratio and gonadal irradiation was observed in childhood cancer survivors in a study by Reulen et al.^59^

Future studies on the effect of space radiation on both fresh and frozen semen samples are needed to assess the possibility of creating a human sperm bank outside the Earth. A study by Wakayama et al.^60^ analyzed the effect of space radiation on mouse freeze-dried spermatozoa stored for almost six years on the International Space Station. The sperm DNA and fertility were not affected after the storage outside the Earth compared to control preserved on Earth, and the current data show the possibility of storing freeze-dried spermatozoa for more than 200 years in space^60^.

Among potential priorities identified by Jain et al.^43^ for future research regarding reproductive aspects of space flight were topics similar to those covered in this systematic review. Additionally, the effect of space travel on libido and the possibility of pregnancy and birth in space were proposed^43^. Results of this systematic review highlight the need to focus more on both female and male astronauts’ reproductive health in future research, as only 16 studies were found during the literature search, and many more research questions related to reproductive health in female and male astronauts still need to be answered.

There are several limitations of this systematic review. The main limitation is the few included studies and the wide range of reproductive health parameters they focused on. The small sample sizes, different types of populations (healthy volunteers, astronauts), and different methodologies need to be considered when comparing or generalizing the results. The limited number of studies addressing these health concerns underscores the imperative need for future research dedicated to reproductive health in both female and male astronauts.

## Methods

### Eligibility criteria for selecting studies

A systematic review of the effect of space travel or its simulation, e.g., bed rest studies, microgravity simulation, or dry immersion, on reproductive health in human females and males was performed according to the Preferred Reporting Items for Systematic Reviews and Meta-Analyses (PRISMA) guidelines^61^ and according the Space Biomedicine Systematic Review methods (https://sites.google.com/view/sr-methods/home). The search was performed using three databases, PubMed, Web of Science, and Medline Complete, on the 29^th^ of April 2023 by one researcher (MG). The eligibility criteria included: (1) astronauts, space travel, or space simulation; (2) experimental or retrospective studies performed on human participants (animal studies were excluded); (3) description of reproductive health parameters.

### Search strategy and selection process

**Fig. 1:**
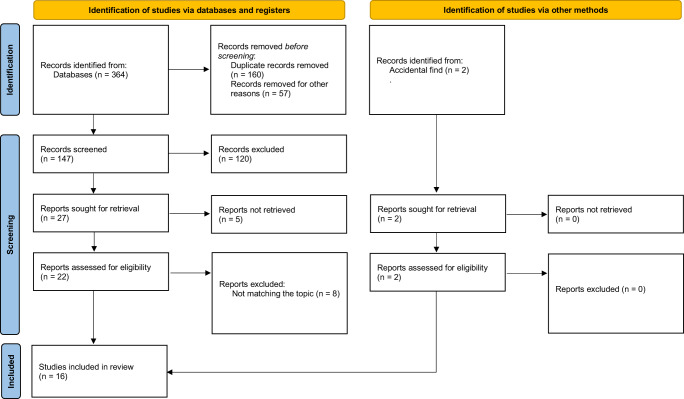
PRISMA flow diagram of the study selection process (template from^61^).

The following terms with Boolean operators were used for the search: (“infertility” OR “birth outcomes” OR “amenorrhea” OR “menstrual” OR “menstrual cycle” OR “follicular phase” OR “luteal phase” OR “menstruation” OR “ovarian cycle” OR “ovulation” OR “anovulation” OR “reproduct*” OR “obstetric*” OR “gynecolog*” OR “maternal” OR “pregnan*” OR “contracept*” OR “prenatal” OR “postpartum” OR “preconception” OR “women’s health” OR “miscarriage” OR “pregnancy loss” OR “menarche” OR “polycystic ovary syndrome” OR “menopause” OR “endometriosis” OR “stillbirth” OR “placental abruption” OR “low birth weight” OR “preterm birth” OR “in vitro fertilization” OR “irregular periods” OR “sperm” OR “testosterone” OR “semen quality” OR “oligospermia” OR “semen” OR “testis” OR “testes” OR “testicular” OR “offspring” OR “reproductive hormone” OR “asthenozoospermia” OR “oligozoospermia” OR “oligoasthenozoospermia” OR “oligoasthenoteratozoospermia” OR “teratozoospermia” OR “spermatogenesis” OR “varicocele” OR “erection” OR “libido” OR “erectile dysfunction” OR “sexual drive”) AND (“space travel” OR “astronaut*” OR “spaceflight” OR “space analogue” OR “cosmonaut*” OR “space simulation” OR “zero gravity” OR “microgravity” OR “hypogravity” OR “low gravity” OR “space radiation”) AND (“human” OR “participant*” OR “women” OR “men” OR “woman” OR “man”) NOT (“review”). The literature search did not exclude any studies published before certain data due to a limited number of scientific studies focused on the analyzed topic as proposed in Ahrari et al.^1^. Studies published until April 2023 were included in this study. Exclusion criteria included animal studies, non-English language, review articles, conference papers, books, and book chapters, and no full-text available. All studies identified in the search were imported into Rayyan systematic review software^62^ to continue the selection process. Studies that did not meet the inclusion criteria (e.g., duplicates, non-English articles, reviews, conference papers, books and book chapters, and animal studies) were excluded by one researcher (MG). The title and abstract of the remaining studies were screened by two researchers (MG, ACP). Any disagreement between researchers was resolved by discussion. After that, the full texts of the included studies were screened to confirm their relevance to the current systematic review. The PRISMA flow diagram summarizes the study selection process (Fig. 1).

### Data collection process and assessment of study quality

Data extraction was performed by two researchers (MG, MB) using a pre-determined form consisting of (i) study characteristics (author, publication year and country, sample characteristics, study setting: Earth/space, and exposure: spaceflight/microgravity/ionizing radiation/bed rest/water submersion/dry immersion); and (ii) analyzed reproductive health parameters and results.

The methodological quality assessment of included studies was performed by one researcher (MG) using the Downs and Black Quality Assessment Checklist^63^. The original checklist consists of 27 questions assessing the quality of reporting, external and internal validity, and statistical power. For this review, 13 items were considered relevant. A similar approach was used in previous studies by Gimunová et al.^64^ and Paludo et al.^65^. A binary score for each question: 0 = no/unable to determine, 1= yes was used. The final score (in %) was classified as follows: <45.4% “poor” methodological quality; 45.5–61.0% “fair” methodological quality”; and >61.0% “good” methodological quality^26^. The quality assessment was not used to exclude any study.

Additionally, as recommended in Space Biomedicine Systematic Review Methods, the Bed rest studies transferability was assessed by the recommended tool (https://sites.google.com/view/sr-methods/guides/bed rest-transferability) used in a previous systematic review by Winnard et al.^66^. The methodological quality of bed rest studies was assessed by one researcher (MG) considering eight questions comparing the study design with “ideal design” resulting in a total score between 0 to 8 points.

### Reporting summary

Further information on research design is available in the Nature Research Reporting Summary linked to this article.

### Supplementary information

Supplementary Table 1. Study quality assessment

Reporting Summary

## Data Availability

The original studies presented in the systematic review are included in the article; further inquiries can be directed to the corresponding author.
