# Supplementary material for: The effect of space travel on human reproductive health: a systematic review
Source: NPJ Microgravity. 2024 Jan 18;10:10. doi: 10.1038/s41526-024-00351-1 (PMC10796912; doi:10.1038/s41526-024-00351-1)
Supplement: Supplementary file 1 — Supplementary Table 1. Study quality assessment [file 41526_2024_351_MOESM1_ESM.pdf]

The Effect of Space Travel on Human Reproductive Health: A Systematic Review

Marta Gimunová[1*], Ana Carolina Paludo[2], Martina Bernaciková[1], Julie Bienertova-Vasku[1]

[1] Department of Physical Activities and Health Sciences, Faculty of Sports Studies, Masaryk University, Brno, Czech Republic

[2] Department of Sport Performance and Exercise testing, Faculty of Sports Studies, Masaryk University, Brno, Czech Republic

[*] Corresponding author, gimunova@fsps.muni.cz

Supplementary Table 1. Study quality assessment.

		Little et al., 1987	Belavý et al., 2012	Boada et al., 2020	Cho et al., 2019	Strollo et al., 2006	Tomilovskaya et al., 2021	Zhou et al., 2016	Gorbacheva et al., 2023	Smorawinski et al., 2001	Zwart et al., 2022	Zachwieja et al., 1999	Liang et al., 2012	Kumar et al., 2013	Ikeuchi et al., 2005	Loder et al., 2006	Smith et al., 2012
**Q1**	**Hypothesis/aim/objective clearly described**	0	0	1	1	1	1	0	0	1	0	1	0	0	0	1	1
**Q2**	**Main outcomes in Introduction or Methods**	1	1	1	1	1	1	1	1	1	1	1	1	1	1	1	1
**Q3**	**Patient characteristics clearly described**	1	1	1	1	1	1	1	1	1	1	1	1	1	1	1	1
**Q6**	**Main findings clearly described**	1	1	1	1	1	1	1	1	1	1	1	1	1	1	1	1
**Q7**	**Estimates of random variability provided for main outcomes**	1	1	1	1	1	1	1	1	1	1	1	1	1	1	1	1
**Q10**	**Probability values reported for main outcomes**	1	1	UD	0	0	1	1	0	0	1	1	1	1	1	1	1
**Q11**	**Subjects asked to participate were representative of source population**	UD	UD	UD	UD	UD	UD	0	UD	UD	UD	UD	UD	1	UD	UD	UD
**Q12**	**Subjects prepared to participate were representative of source population**	1	UD	UD	UD	UD	UD	UD	UD	UD	1	UD	UD	UD	UD	1	UD
**Q16**	**Any data dredging clearly described**	1	1	1	1	1	1	1	1	1	1	1	1	1	1	1	1
**Q18**	**Appropriate statistical tests performed**	1	1	1	1	1	1	1	1	1	1	1	1	1	1	1	1
**Q20**	**Outcome measures were reliable and valid**	1	1	1	1	1	1	1	1	1	1	1	1	1	1	1	1
**Q26**	**Losses to follow-up taken into account**	UD	UD	UD	UD	UD	UD	UD	UD	UD	UD	UD	UD	UD	UD	UD	UD
**Q27**	**Sufficient power to detect treatment effect at significance level of 0.05**	0	0	0	0	0	0	0	0	0	0	0	0	0	0	0	0
	**Total score**	9	8	8	8	8	9	8	7	8	9	9	8	9	8	10	9
	**Maximum rate possible**	11	10	9	11	11	11	11	10	10	11	10	10	11	10	11	10
	**Kennelly score (%)**	81,8	80,0	88,9	72,7	72,7	81,8	72,7	70,0	80,0	81,8	90,0	80,0	81,8	80,0	90,9	90,0
